# Insights on the UV-Screening Potential of Marine-Inspired Thiol Compounds

**DOI:** 10.3390/md22010002

**Published:** 2023-12-19

**Authors:** Alessia Luccarini, Annalisa Zuccarotto, Roberta Galeazzi, Camilla Morresi, Mariorosario Masullo, Immacolata Castellano, Elisabetta Damiani

**Affiliations:** 1 Department of Life and Environmental Sciences, Polytechnic University of Marche, 60131 Ancona, Italy; a.luccarini@pm.univpm.it (A.L.); r.galeazzi@univpm.it (R.G.); c.morresi@staff.univpm.it (C.M.); 2 Department of Biology and Evolution of Marine Organisms, Stazione Zoologica Anton Dohrn, 80121 Naples, Italy; annalisa.zuccarotto@szn.it; 3 Department of Medical, Movement and Wellbeing, University of Naples “Parthenope”, 80133 Naples, Italy; mario.masullo@uniparthenope.it; 4 Department of Molecular Medicine and Medical Biotechnology, University of Naples “Federico II”, 80121 Naples, Italy

**Keywords:** photoprotection, ovothiols, 5-thiohistidine, marine-inspired UV filters, eco-friendly sunscreens

## Abstract

One of the major threats to skin aging and the risk of developing skin cancer is excessive exposure to the sun’s ultraviolet radiation (UVR). The use of sunscreens containing different synthetic, organic, and inorganic UVR filters is one of the most widespread defensive measures. However, increasing evidence suggests that some of these compounds are potentially eco-toxic, causing subtle damage to the environment and to marine ecosystems. Resorting to natural products produced in a wide range of marine species to counteract UVR-mediated damage could be an alternative strategy. The present work investigates marine-inspired thiol compounds, derivatives of ovothiol A, isolated from marine invertebrates and known to exhibit unique antioxidant properties. However, their potential use as photoprotective molecules for biocompatible sunscreens and anti-photo aging formulations has not yet been investigated. Here, we report on the UVR absorption properties, photostability, and in vitro UVA shielding activities of two synthetic ovothiol derivatives, 5-thiohistidine and iso-ovothiol A, by spectrophotometric and fluorimetric analysis. We found that the UVA properties of these compounds increase upon exposure to UVA and that their absorption activity is able to screen UVA rays, thus reducing the oxidative damage induced to proteins and lipids. The results of this work demonstrate that these novel marine-inspired compounds could represent an alternative eco-friendly approach for UVR skin protection.

## 1. Introduction

In the Anthropocene era, the thinning of the ozone layer has led to the excessive exposure of terrestrial and marine organisms to the sun’s ultraviolet radiation (UVR) which in humans can induce skin damage, accelerate skin aging, and increase the risk of developing skin cancer. Although the UVC rays (<280 nm) and most of the UVB ones (280–320 nm) are shielded by stratospheric ozone, ~5% of UVB and ~90% of UVA (320–400 nm) rays reach the Earth. The deleterious effects of UVR on the skin, including skin aging and dermal pathologies, are cumulative, irreversible, and currently responsible for 90% of skin aging as well as skin cancer development [1]. Consequently, among the recommendations from the World Health Organization is the use of sunscreens. Sunscreens prevent the development of UVR-mediated skin processes e.g., aging and wrinkle formation, and protect against skin cancer [2,3]. Nowadays, consumers are increasingly aware of the origin (synthetic or natural) and eco-sustainability of their personal care products, and recent concerns have been raised on the potential eco-toxicity of some sunscreens to the marine environment and human health [4,5,6]. Indeed, sunscreens containing certain UV synthetic filters (benzophenone-3, octylmethoxycinnamate) are currently banned from sale and distribution in Hawaii and other USA nations for their suspected harm to coral reefs and marine life [7]. For example, several organic UV filters are known to induce endocrine disruption, hepatotoxicity, mutagenicity, and systemic toxicity in various organisms [8]. Therefore, turning to nature to look for natural products to replace synthetic chemicals is a growing trend in the pharmaceutical/cosmeceutical sector. In this context, photoprotective compounds derived from marine environments and extracted either from their natural sources or produced by engineering yeast or other microorganisms, such as bacteria and algae [9] represent an attractive strategy. Indeed, marine organisms living in the photic zone of seas and constantly exposed to changes in light intensity and spectral composition have evolved the ability to use solar radiation for survival but also to protect themselves from UVR-induced damage, e.g., by producing photoprotective molecules [9,10,11,12]. Hence, the marine environment represents a yet untapped source of naturally occurring UVR screening agents that could be used as eco-friendly and safer alternatives to synthetic UV filters [11,12]. For example, mycosporine-like amino acids (MAAs), like shinorine, palythine, asterina, and scytonemin are well-known UV-absorbing/UV-screening compounds produced by cyanobacteria, microalgae, and macroalgae [11,12].

This work aimed to characterize the UV-screening properties of novel marine-inspired compounds to develop natural-based sunscreens and personal-care products that could be both safer for human use and more environmentally friendly. Our attention focused on sulfur-containing histidine compounds, named ovothiols, known to be produced by several marine invertebrates, like the sea urchin *Paracentrotus lividus*, bacteria (i.e., *Erwinia tasmaniensis*), and microalgae, especially diatoms belonging to stramenopiles, like *Skeletonema marinoi* and *Phaeodactylum tricornutum* [13,14,15,16,17]. These compounds occur in three differently methylated forms (A, B, and C) and are characterized by unique redox properties suggestive of numerous cellular functions [14,16,17,18,19]. They possess unusual antioxidant properties due to the peculiar position of the sulfhydryl group on the imidazole ring of histidine, which confers ovothiols remarkable reactivity towards reactive oxygen species (ROS) [14,20,21,22]. The pKa of the thiol group of ovothiol A is much lower (~1.4) than that of glutathione (GSH) (~8.7). On the other hand, the disulphide of ovothiol A is less stable than the disulphide of GSH (GSSG). The two-electron redox potential of ovothiol A is considerably more positive (E = −0.09 V vs. standard hydrogen electrode) than that of GSH (E = −0.26 V) [14]. Therefore, the more reactive ovothiol A and the more reductive GSH have been proposed to cooperate to protect cells against hydrogen peroxide-induced damage [23]. According to this model, ovothiol A would first reduce peroxides by nucleophilic attack, the resulting ovothiol A disulphide would subsequently be reduced by GSH, and finally, the resulting GSSG would be recycled by NAD(P) H-dependent glutathione reductases [23]. Recent investigations also point to the anti-oxidant and anti-inflammatory activities of ovothiols in in vitro models of human endothelial dysfunction and inflamed keratinocytes, in an in vivo model of liver fibrosis, and in ex vivo human inflamed skin tissues [24,25,26,27]. In addition, the regulation of ovothiol biosynthesis has been associated with environmental exposure to metals and toxins present in seawater in *P. lividus* embryos and larvae [18], to high light in the diatom *S. marinoi* [28], and to UVR in the sea anemones *Nematostella vectensis* [29]. These findings, together with the recent discovery of ovothiols in the lens of some fish [30], are suggestive of their potential photoprotective activities. In addition, ergothioneine, another natural product belonging to the class of thiohistidines [31], has been shown to possess dermo-protective properties in UVA-irradiated keratinocytes, although its UV-absorbing properties have not been fully explored [32].

In this study, we investigated the absorbance profile of ovothiol derivatives of chemical synthesis, 5-thiohistidine (5-thio) and iso-ovothiol A (iso-ovoA), inspired by their natural counterparts [33] when exposed to UVA light. The only structural difference between the natural ovothiol A and iso-ovoA is that the latter is characterized by a methyl group at position τ of the imidazole ring and not at position π of the imidazole ring, as in ovothiol A. Our study shows that 5-thio and iso-ovoA display a significant UVA absorption capacity. Their UV shielding properties were confirmed using different spectrophotometric and fluorometric assays, which showed their potential to inhibit UV-induced oxidation of relevant biological macromolecules (lipids and proteins). These results could lay the foundation for the development of biocompatible, marine-inspired UV skin protectants that could simultaneously address issues related to the impact of synthetic UV filters on the health of marine habitats.

## 2. Results

### 2.1. Absorption Spectra of Oxidized and Reduced Forms of Marine-Inspired Thiol Compounds

**Figure 1:**
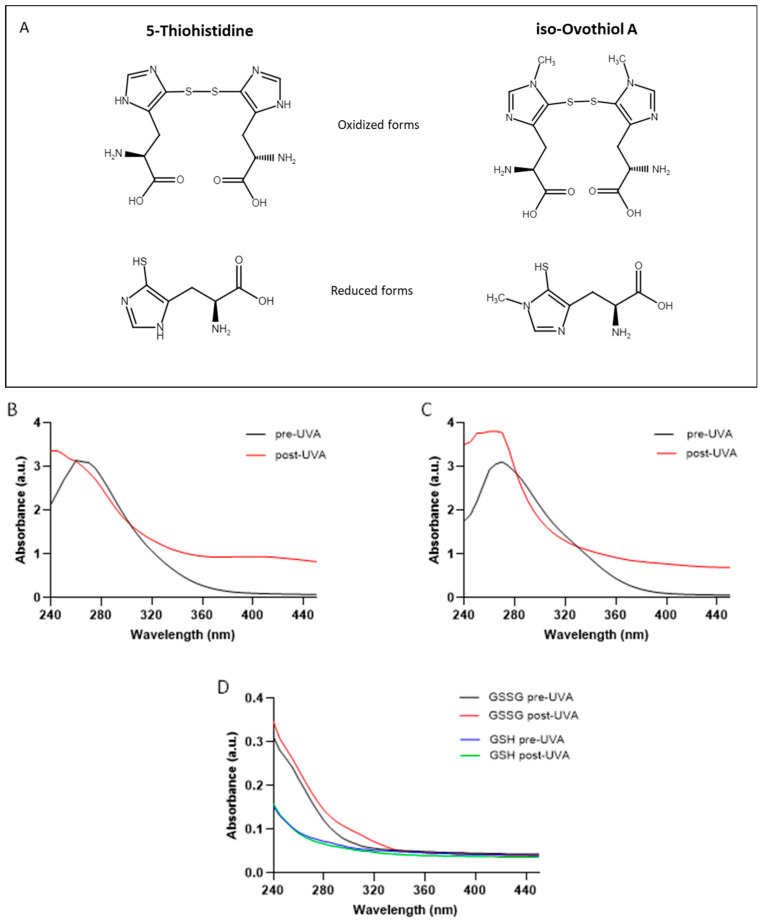
(**A**) Chemical structure of the oxidized and reduced forms of 5-thio and iso-ovoA. (**B**) Optical absorption spectra of 5-thio (0.9 mM) and (**C**) of iso-ovoA (0.9 mM) in their oxidized form, not exposed (black line) and exposed (red line) to 20 min UVA (540 kJ/m^2^). (**D**) Optical absorption spectra of GSSG (black and red lines) and GSH (blue and green lines) forms (0.9 mM) before (black and blue lines) and after 20 min UVA exposure (red and green lines).

The disulphide form of 5-Thio and iso-ovoA (Figure 1A) were subjected to 20 min UVA exposure and their absorbance spectra were monitored. The exposure time of 20 min, equivalent to ~540 kJ/m^2^, was chosen since longer exposure times did not induce further changes in absorbance spectra. As shown in Figure 1B,C, UVA exposure induces a change in the absorption spectrum of both molecules. In the absence of treatment, both 5-thio and iso-ovoA display a characteristic peak of absorbance around 254/260 nm, typical of the natural counterparts [34]. After UVA irradiation, this peak is no longer clearly observed. On the contrary, a significant increase in absorbance starting from 320 nm can be noted in both compounds compared to those not exposed to UVA. To compare these results with other well-known thiol compounds, the same analysis was performed with the reduced and oxidized glutathione (GSH and GSSG, respectively) exposed to UVA under identical conditions (Figure 1D). Besides the difference observed in the spectra of GSSG and GSH in the absence of UVA irradiation, no remarkable differences were observed after UVA irradiation for both GSSG and GSH, compared to not irradiated solutions, indicating the stability of both compounds under UVA irradiation.

To understand if the change in the absorption spectra of 5-thio and iso-ovoA, following UVA exposure, was due to a change in the redox state of the compounds, we performed the Ellman assay. This assay implies the use of the 5,5-dithio-bis-(2-nitrobenzoic acid) (DTNB) reagent, which should reduce the disulphide bond, giving rise to the yellow colour of the TNB^2−^ dianion measurable at 412 nm. Table 1 shows that when both compounds (5-thio and iso-ovoA) are mixed with DTNB, the reaction displays an absorbance value around zero, thus confirming their oxidized form. On the contrary, upon UVA exposure of the two compounds, there is a significant increase in absorbance at 412 nm in the presence of DTNB, indicative of the reduction of 5-thio and iso-ovoA. To validate this assay, GSSG and GSH were also tested with the same assay, confirming their respective oxidized/reduced states in the absence of UVA irradiation. However, under UVA irradiation, both spectra showed no differences compared to non-irradiated conditions. These results suggest that UVA exposure induces the reduction of 5-thio and iso-ovoA, but not of GSSG.

### 2.2. Comparison of UV Absorption Spectra of Chemically Reduced 5-thio and iso-ovoA

**Figure 2:**
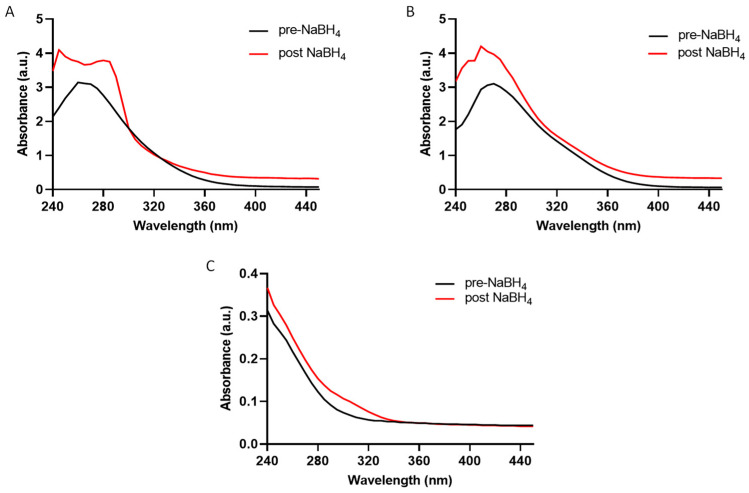
Optical absorption spectra of equimolar concentrations (0.9 mM) of 5-thio (**A**), iso-ovoA (**B**), and GSSG (**C**) pre- and post-reduction with NaBH_4_.

To further confirm that under UVA exposure, the thiol compounds undergo reduction, a sodium borohydride (NaBH_4_) reagent was used to investigate the transition of 5-thio and iso-ovoA from the oxidized to the reduced form. As shown in the following figure, both 5-thio (Figure 2A) and iso-ovoA (Figure 2B) undergo an increase in absorbance after reduction with NaBH_4_, similar to the change in the spectrum profile obtained in Figure 1B,C. GSSG spectra pre- and post-NaBH_4_ (Figure 2C) support the previously obtained results, i.e., no change in absorbance in the two conditions, confirming the greater resistance of the disulphide bond of GSSG to reduction (Figure 1D).

The Ellman assay was also repeated on 5-thio, iso-ovoA, and GSSG pre- and post-reduction with NaBH_4_ to confirm the reduction of the compounds (Table 2). While in the absence of NaBH_4_ treatment, the absorbance value at 412 nm for the two marine-inspired thiol compounds was around zero, as would be expected due to their disulphide form, the absorbance value at 412 nm significantly increased post-NaBH_4_ treatment, thus demonstrating that UVA exposure leads to the reduced forms of these compounds, since the behaviour using a chemical reducing agent is similar. However, in the case of GSSG there was no change in absorbance, indicating the stability of the disulphide bond in this compound.

### 2.3. DFT Quantum Mechanical Calculations of Disulphide Bonds

DFT calculations carried out on the thiol compounds were aimed at calculating the bond dissociation energies (BDEs) of the disulphide bond S-S (Table 3). The results obtained confirm that the BDE of the disulphide bond of oxidized 5-thio and iso-ovoA are longer and weaker than the disulphide bond in GSSG, thus supporting the results reported above concerning the greater resistance of GSSG to cleavage by UVA and to reduction by chemical agents. Moreover, a slight difference in the length and strength of the disulphide bond is observed between the thiohistidine derivatives, showing that the S-S bond of the natural counterpart, ovothiol A, is more stable compared to 5-thio, which in its turn is more stable than iso-ovoA.

### 2.4. Shielding Effect of Marine-Inspired Thiol Compounds against UVA-Induced Oxidative Modification of Proteins

**Figure 3:**
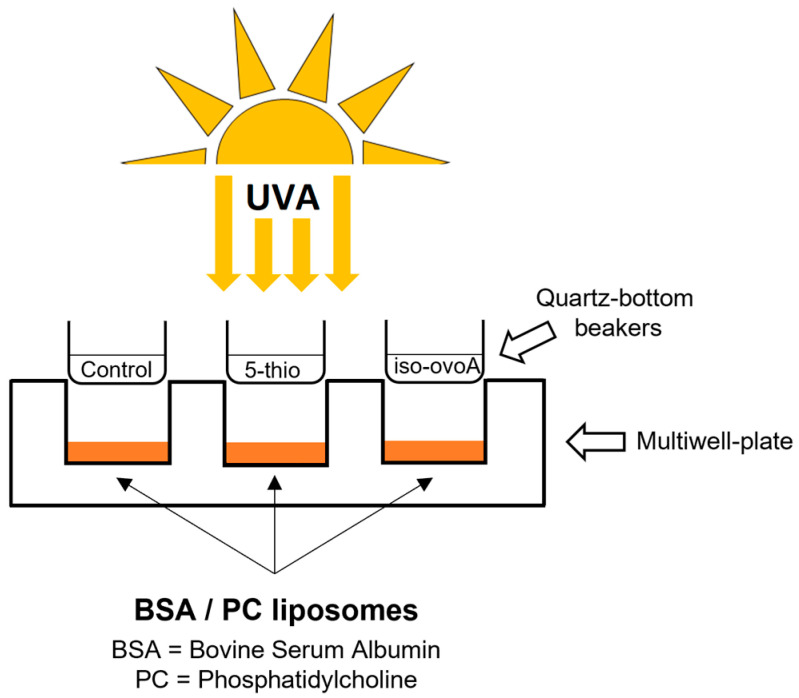
Representative scheme of UVA exposure using marine-inspired thiol compounds as shielding agents to prevent photo-oxidative stress of different macromolecules.

**Figure 4:**
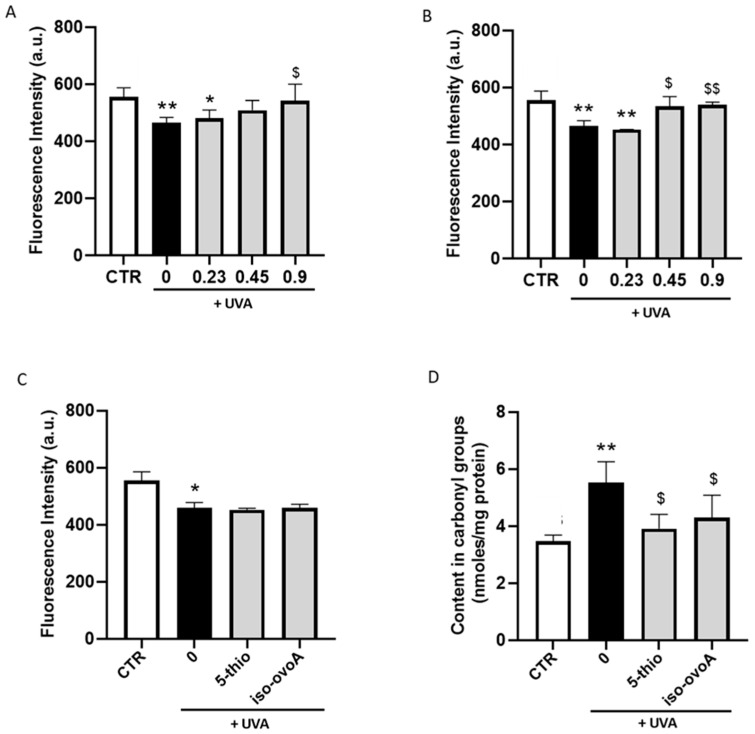
Photo-oxidative damage in BSA in the presence of 5-thio and iso-ovoA used as shielding agents. Fluorescence intensity of Trp in the presence or absence of pre-irradiated compounds at different concentrations of 5-thio (**A**) and iso-ovoA (**B**). (**C**) Fluorescence intensity of Trp in the presence or absence of 0.9 mM non-pre-irradiated compounds. (**D**) Content in carbonyl groups after 20 min UVA exposure using pre-irradiated compounds (0.9 mM). All exposures were performed at 20 min UVA irradiation (540 kJ/m^2^). Error bars represent ± S.D. * *p* < 0.05; ** *p* < 0.001 vs. control (CTR); $ *p* < 0.05, $$ *p* < 0.001 vs. 0 (exposed to UVA).

To investigate the shielding effect of the two marine-inspired thiol compounds, we used the experimental setup reported in Figure 3. As a starting point, different concentrations (0.23, 0.45, and 0.9 mM) of pre-irradiated 5-thio and iso-ovoA compounds were used to determine their possible shielding effect on bovine serum albumin (BSA) after UVA exposure. Figure 4 clearly shows that, in the absence of the compounds, after 20 min UVA exposure, tryptophan (Trp) fluorescence of BSA decreased, thus indicating a change in the conformational status of the protein upon UVA exposure. On the contrary, in the presence of 0.9 mM 5-thio (Figure 4A) and in the presence of both 0.45 and 0.9 mM iso-ovoA, the degree of Trp fluorescence intensity of BSA was significantly restored to the levels of the unexposed control (Figure 4B).

Based on the above results, the concentration of 0.9 mM for 5-thio and iso-ovoA was selected for subsequent experiments. The same experiments were performed using both 5-thio and iso-ovoA not pre-exposed to UVA, i.e., in their oxidized form. The results shown in Figure 4C do not highlight significant differences in Trp fluorescence of BSA in the presence of the compounds compared to their absence upon UVA irradiation. This indicates that the compounds not pre-exposed to UVA, i.e., in their oxidized form, do not shield, hence they do not prevent UVA-induced oxidative modifications of proteins. Moreover, the degree of protein carbonyl formation in BSA, which represents a measure of the oxidized status of proteins, increased after 20 min UVA exposure; however, in the presence of the pre-irradiated compounds, i.e., in their reduced form, the levels significantly decreased compared to the control exposed to UVA (Figure 4D).

### 2.5 Shielding Effect of Marine-Inspired Thiol Compounds against UVA-Induced Lipid Peroxidation

**Figure 5:**
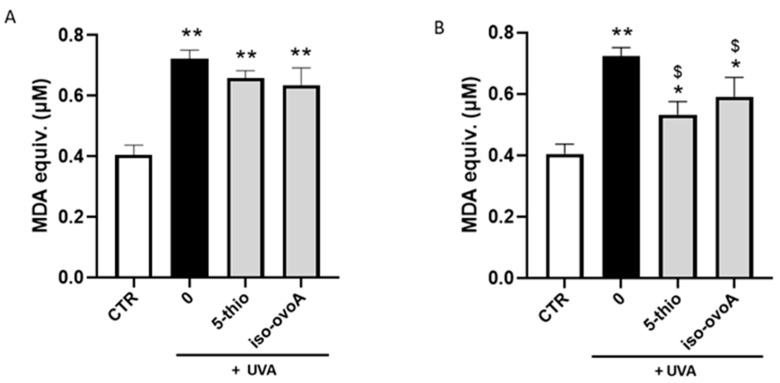
UVA-induced lipid peroxidation in PC liposomes in the presence or absence of marine-inspired thiol compounds. Iso-ovoA and 5-Thio (0.9 mM) were used as shielding agents in the oxidized (**A**) and in the reduced forms (**B**), exposed to 20 min UVA irradiation (540 kJ/m^2^). Error bars represent ± S.D. * *p* < 0.05, ** *p* < 0.001 vs. control (CTR); $ *p* < 0.05 vs. 0 (exposed to UVA).

To evaluate the potential shielding effects of marine-inspired thiol compounds on lipid peroxidation, PC liposomes were exposed to 20 min UVA using the experimental setup shown in Figure 3, in the presence or absence of 0.9 mM 5-thio and iso-ovoA non-irradiated (oxidized forms) (Figure 5A) and pre-irradiated (reduced forms) (Figure 5B). The thiobarbituric acid reactive (TBARS) assay was used to detect the level of malondialdehyde (MDA) and measure lipid peroxidation. As illustrated in Figure 5, the TBARS levels increased almost two-fold upon UVA exposure; however, in the presence of not pre-irradiated compounds, no significant differences in the extent of lipid peroxidation can be observed compared to the control (0) (Figure 5A). On the contrary, using the reduced (pre-irradiated) forms of the marine-inspired thiol compounds (Figure 5B), the levels of TBARS significantly decreased compared to the UVA-exposed control (0).

## 3. Discussion

In this work, we have presented the first chemical characterization of the UVA absorption properties and UVA shielding effects of novel synthesized histidine derivatives inspired by the chemical structure of marine natural products, commonly named ovothiols. These compounds are characterized by an aromatic imidazole ring, able to absorb UV rays at 254/280 nm with a peculiar absorption spectrum in the UVB–UVA range [9]. In addition, they exhibit unique redox properties thanks to the peculiar position of the thiol group on the imidazole ring, which confers them a strong antioxidant potential [14,23]. These features indicate that ovothiols may display UVA-protecting properties, resembling the behaviour of MAAs, which are currently considered the strongest UVA-absorbing compounds in nature [11,12,35], due to their ability to absorb light both in the UVA (315–400 nm) and UVB (280–315 nm) range and to their high molar extinction coefficient [11,12]. Since obtaining ovothiols from the most abundant natural source e.g., sea urchin eggs [34], cannot be considered an eco-sustainable strategy, recent efforts have been devoted to developing engineered microorganisms aimed at their biosynthesis [17] and to screening the chemical and biological properties of synthetic derivatives to compare their activities with natural counterparts for further industrial development.

Here, we first show that marine-inspired thiols, 5-thio and iso-ovoA, provided in their oxidized forms which represent the more stable form of these highly reactive thiols, undergo a reduction of the disulphide bond upon UVA exposure, contextually exhibiting a major absorption factor in the UVA range (320–400 nm), a property that makes them interesting as potential UV filters for sunscreens. Our data confirm that for 5-thio and iso-ovoA, as occurred for the natural counterpart ovothiol A, the disulphide bond is less stable compared to the disulphide of the most ubiquitous thiol, GSSG, which is resistant to homolytic cleavage induced by UVA and to reduction by chemical agents. The differences in the stability of the disulphide forms are reflected by the length of the S-S bonds. Indeed, the length of the S-S bond of the natural product ovothiol A is greater compared to that of GSSG, likely due to the repulsion of the two imidazolic rings. Among 5-thiohistidine derivatives, the S-S bond of 5-thio is slightly weaker compared to ovothiol A, probably due to stabilization by the methyl group on the imidazolic ring of ovothiol A. On the contrary, the S-S bond of iso-ovoA is even weaker than that of 5-thio, likely due to the repulsion of the methyl groups in position τ of the imidazolic rings, which in this case are closer compared to their position in ovothiol A.

Curiously, our data show that both 5-thio and iso-ovoA in their reduced form, and not in the oxidized one, are able to effectively shield UVA, reaching an absorption value of 1, which is likely responsible for preventing the in vitro UVA-induced oxidative damage to proteins like BSA and lipids in the form of liposomes. The reasons for the difference between the reduced and oxidized form remains obscure. However, we can hypothesize that the intermolecular interactions between the thiohistidine compounds change in the two states and are responsible for the different absorption behaviours. For example, one can imagine more structural constraints for the oxidized form compared to the reduced one. On the other hand, the reduced forms could undergo resonance hybrids.

In conclusion, our data indicate that the reduced compounds display the peculiar characteristics of UV filters and need to be tested in in vitro cellular systems to evaluate their potential UVA protection properties in contact with cells to promote their use in eco-safety sunscreen formulations. Interestingly, previous toxicity tests on the natural ovothiol A and 5-thio proved no toxicity of these compounds, at least up to 200 µM [24,27]. Generally, the compounds are administered in the oxidized forms to cell cultures; however, they are known to be reduced by the intracellular environment, thus acting as antioxidants [23,24]. In this regard, it is interesting to highlight that both 5-thio and the natural ovothiol A have been reported to induce the translocation of the nuclear factor erythroid 2-related factor 2 (Nrf2) into the nucleus, thus activating an anti-inflammatory response in human keratinocytes [27]. A similar behaviour has been reported for some MAAs, like shinorine, in primary skin fibroblast cells [11,35]. On the other hand, they should not represent a menace to the environment, since several marine organisms use the natural compounds as protective molecules against different environmental stressors [18,28]. Interestingly, the recent discovery of ovothiols in the lenses of fish is predictive of specialized UVA protection properties in the eyes [36]. This represents a very interesting finding, considering that aged and cataractous human lenses are more vulnerable to UVA light than middle-aged lenses [37]. Therefore, future studies on these nature-derived marine compounds could also shed new light on their potential use as photo-protectants to prevent cataracts and age-related pathologies of the eyes. Future studies will also be fundamental in comparing the UVA protective activities of these compounds with other marine-derived natural products, like MAAs, to establish their potential efficacy in developing sunscreens.

## 4. Materials and Methods

### 4.1. Reagents

L-α-phosphatidylcholine (PC) (P2772), Bovine Serum Albumin (BSA) (A6003), Trichloroacetic acid (TCA), and butylated hydroxytoluene (BHT), as well as all other reagents and solvents unless otherwise stated, were purchased from Merck (Merck KGaA, Darmstadt, Germany). 5,5-dithio-bis-(2-nitrobenzoic acid) (DTNB) was from Cayman Chemical Company (Cayman Chemical Company, Michigan, USA). Iso-ovoA and 5-thio were kindly donated by Jean Claude Yadan, Tetrahedron Company (Paris, France).

### 4.2. UV-Vis Spectrophotometric Measurements

Stock solutions (10 mM) of the synthetic, nature-derived marine compounds were prepared in deionized water. Appropriate amounts of phosphate buffered saline (PBS) were then added to reach the final concentration of 0.9 mM for 5-thio and iso-ovoA. The solutions (200 μL) were then transferred to a UV-transparent 96-multi-well plate and the absorption spectrum between 240–450 nm was determined on a multiplate reader (BIO-TEK Synergy HT), before and after 20 min UVA exposure.

### 4.3. UVA Exposure

For UVA exposure, the 96-multi-well plate set up as described above (paragraph 4.2), was covered with a 2 mm-thick quartz slab to prevent any evaporation during irradiation. For exposure of BSA or PC liposomes (see Section 4.7, Section 4.8 and Section 4.9), these were transferred to a 24-multi-well plate and exposed to UVA in the presence or absence of the reduced forms of 5-thio and iso-ovoA. To determine the UV-shielding effect of the thiol compounds, solutions (500 μL) of these compounds were transferred into custom-made quartz-bottom beakers of exactly the same dimension as the wells of a 24-multi-well plate and placed on top of each well (see Figure 5). The multi-well plate was placed on a brass block embedded in ice at a distance of 20 cm from a light source consisting of a Philips Home Solarium sun lamp (model HB 406/A; Philips, Groningen, Holland) equipped with a 400 W ozone-free Philips HPA Lamp, UV type 3, delivering a flux of 45 mW/cm^2^ between 300 and 400 nm. The dose of UVA and emission spectrum were measured as reported previously with a UV Power Pack Radiometer (EIT Inc. Sterling, VA, USA) [38]. For all experiments, unless otherwise stated, samples were exposed to 20 min UVA (~540 kJ/m^2^) based on preliminary time-course experiments. As positive control, an empty beaker was used, while for the negative control, the samples were not exposed to UVA.

### 4.4. Ellman’s Assay

DTNB reagent was reconstituted by adding 500 μL DMSO to make 20 mM stock solutions. In a 96-multi-well plate, 8 μL of 1 mM DTNB working solutions was added to both 200 μL 5-thio and iso-ovoA (0.9 mM), which were then exposed or not to UVA. The absorbance of each sample was then read at 412 nm on a microplate reader. DTNB reagent was also added to GSH and GSSG glutathione forms (0.9 mM), used as a control [24].

### 4.5. Sodium Borohydride Assay

Sodium borohydride (NaBH_4_) 10 mM stock solution in PBS was used as a reducing agent. Solutions (18 μL) of 5-thio and iso-ovoA (0.9 mM), were transferred into wells of a 96-multi-well plate to which an equimolar quantity of NaBH_4_ was added (final volume = 200 μL). Solutions of the marine-inspired thiol compounds without NaBH_4_ were also transferred into the same plate as control. The UV-Vis absorption spectrum between 240–450 nm was then analysed for all the samples on the microplate reader. The same procedure was also performed with GSSG.

### 4.6. Computational Calculations

To determine the homolytic BDEs of the compounds tested, Gaussian 16 [39] was used for all DFT calculations with a default ultra-fine grid for all numerical integrations. The equilibrium geometries of reactants (RS-SR) and the free radicals produced (RS^●^) were fully optimized by employing the hybrid density functional B3LYP with the 6–31 G (d,p) basis set, since previous studies confirmed the accuracy of this functional and basis set for optimization and frequency calculations of this class of organic compounds [40,41,42,43]. All optimizations were checked for convergence to an energy minimum, which included checking for proper termination flags from Gaussian and ensuring the resulting structure had no imaginary vibrational frequencies. Standard thermodynamic parameters at 298 K, including zero-point energy correction (ZPE), were obtained by vibrational frequency calculations. The varied conformers were fully optimized to find the energy minima. Through comparisons of these energy minima, the global energy minimum (no imaginary frequency) was selected as the research object.

The BDE is the total energy required for homolytic cleavage of a specific bond, in this case, the disulphide bond (S-S) of the oxidized thiol (RS-SR) compounds. The BDE is thus the enthalpy change of the following reaction:

RS-SR → 2RS^●^

The BDE calculations were performed according to the protocol of Wright [44] which is widely used in the literature [45]. It can be expressed numerically as:

BDE(RS-SR) = 2[E(RS^●^) + EZP(RS^●^)] − E(RS-SR) – EZP(RS-SR)

where E is molecular energy and EZP is the zero-point energy.

### 4.7. Protein Carbonyl Group Content Determination

The protein samples were prepared by dissolving 3 mg/mL of BSA in 50 mM PBS, 0.1 mM ethylenediaminetetraacetic acid (EDTA), pH 7.4. Then, 1 mL of this solution was transferred into each well of the 24-multiwell-plate and exposed to UVA as described in Section 4.3 [46]. After UVA exposure, protein oxidation was assessed using the method of Levine et al., which uses the reaction of 2,4-dinitrophenylhydrazine (DNPH) with the carbonyl groups of oxidized proteins [47]. DNPH (500 μL of 20 mM solution in 2.5 M HCl) was added to 500 μL of each sample and incubated for 1 h at room temperature under continuous shaking. The remaining volume (500 μL) of each sample was used for the corresponding blank after the addition of 500 μL 2.5 M HCl without DNPH and subjected to the same 1 h incubation. The protein was then precipitated by the addition of 500 μL of 20% trichloroacetic acid (TCA) (*w*/*v*) and centrifuged at 3000× *g* for 10 min, then the supernatant was discarded. The residual pellet was washed by adding 500 μL of ethanol-ethyl acetate (1:1 *v*/*v*) followed by centrifugation at 3000× *g* for 10 min. This process was repeated twice before the residual pellet was dissolved in 1 mL 6 M guanidine HCl (pH 6.5). Absorbance at 370 nm was measured spectrophotometrically (Spectrophotometer UVIKON 941 Plus) and the content of carbonyl groups was evaluated using a molar absorption coefficient of 22.000 M^−1^ cm^−1^. The obtained results are reported as the content of carbonyl groups in nmol/mg of protein.

### 4.8. Intrinsic Tryptophan Fluorescence

The protein samples were prepared by dissolving 10 mg/mL of BSA in PBS, then 500 μL of this solution was transferred to each well of a 24-multi-well plate. After UVA exposure, each protein sample was diluted 1:500 in PBS before detection of tryptophan fluorescence. Fluorescence measurements were made at the right angle in 10 mm × 2 mm dual pathlength quartz cuvettes on an LS-55 PerkinElmer fluorescence spectrophotometer (Waltham, MA, USA). An excitation–emission matrix (EEM) was recorded for each sample, at excitation wavelengths of 280 nm and emission wavelengths from 300 to 500 nm. The fluorescence intensity was measured between 330–340 nm. The excitation and emission slits were respectively set to 5–4 nm.

### 4.9. Liposome Preparation and Evaluation of Lipid Peroxidation

Liposome preparation was obtained as reported previously [38]. Briefly, the desired amount of PC in chloroform was added to a glass test-tube kept in an ice bath and the solvent was thoroughly evaporated under a stream of nitrogen. The resulting lipid film was dispersed in 1.5 mL of 5 mM PBS, 0.1 mM EDTA, pH 7.4, and vortexed for 1–2 min until a white, homogeneous, opalescent suspension was obtained. The final concentration of PC in the resulting multilamellar liposomal dispersion was 3.5 mM. Each sample was then aliquoted into two parts (700 μL each) and transferred into a 24-multi-well plate and exposed to UVA as described in Section 4.3. As non-illuminated control, 700 μL from each liposomal suspension was kept in the dark on ice. After UVA exposure, the extent of lipid peroxidation was assessed using the thiobarbituric acid (TBA) assay with slight variations [48]. A volume of 2 mL of TBA-TCA-HCl (0.375% *w*/*v* TBA, 15% *w*/*v* TCA, 0.2 M HCl) was added to 600 μL of sample containing BHT (20 mM) to prevent possible peroxidation of liposomes during the TBA assay. Samples were then incubated for 20 min at 95 °C followed by cooling and centrifugation at 5000× *g* for 6 min. For the determination of the aldehydic breakdown products of lipid peroxidation, the absorbance of the supernatant was measured at 535 nm (Spectrophotometer UVIKON 941 Plus). A standard curve of 1,1,3,3-teraethoxypropane was used to quantify the amount of malondialdehyde produced per sample.

### 4.10. Statistical Analysis

All experiments were performed at least three times in duplicate and conducted in different experimental sessions. Data are expressed as the mean ± standard deviation. Statistical analyses were performed using the GraphPad Prism 8.2 software one-way analysis of variance (ANOVA). Values of *p* < 0.05 were considered statistically significant (Tukey’s post hoc multiple-comparison test).

## Figures and Tables

**Table 1:** Absorbance values of DTNB reagent were measured at 412 nm, after reaction with 5-thio and iso-ovoA, before and after 20 min UVA exposure. Both GSSG and GSH were used for comparison. The results are expressed as mean value ± S.D. (n = 3). ** *p* < 0.001 vs. pre-UVA.

	pre-UVA	post-UVA
GSSG	−0.055 ± 0.016	−0.041 ± 0.007
GSH	0.522 ± 0.004	0.481 ± 0.020
5-thio	0.004 ± 0.001	0.939 ± 0.016 **
iso-ovoA	−0.065 ± 0.021	0.601 ± 0.025 **

**Table 2:** Absorbance values from the Ellman assay for 5-thio and iso-ovoA, pre- and post-reduction with NaBH_4_. GSSG was used for comparison. The results are expressed as mean value ± S.D. (n = 3). * *p* < 0.05 vs. pre-NaBH_4_.

	NaBH_4_	
	−	+
5-Thio	0.003 ± 0.004	0.507 ± 0.095 *
iso-OvoA	−0.020 ± 0.028	0.449 ± 0.091 *
GSSG	−0.041 ± 0.057	−0.033 ± 0.056

**Table 3:** Calculated bond dissociation energy (BDE) and bond length for the S-S bond of the oxidized thiol compounds.

Compound (S-S Dimer)	BDE (with ZPE Correction) (kJ/mol)	S-S Bond Length (Å)
iso-ovoA	99.44	2.19
5-thio	111.94	2.20
ovothiol A	115.24	2.15
GSSG	145.46	2.09

## Data Availability

Data are contained within the article.
